# Hypertension Is an Independent Predictor of Multivessel Coronary Artery Disease in Young Adults with Acute Coronary Syndrome

**DOI:** 10.1155/2018/7623639

**Published:** 2018-11-13

**Authors:** Junhua Ge, Jian Li, Haichu Yu, Bo Hou

**Affiliations:** Department of Cardiology, The Affiliated Hospital of Qingdao University, Jiangsu Road 16, 266000 Qingdao, Shandong, China

## Abstract

**Background:**

Risk factors of multivessel coronary artery disease (CAD) among young acute coronary syndrome (ACS) patients remain elusive now.

**Methods:**

This retrospective study analyzed data from 187 consecutive young (age ≤45 years) ACS patients (75 STEMI, 30 NSTEMI, and 72 unstable angina) hospitalized in our hospital from January 2012 to December 2016. Thirty-six young male patients with normal coronary angiography (CAG) findings (no-CAD), who underwent CAG due to suspected chest pain in this period, served as control group. There were 83 patients with single-vessel disease (SVD) and 104 patients with multiple-vessel disease (MVD) among ACS patients. Patients were followed up for a mean of 267±124 days by clinical visit or telephone calls.

**Results:**

All included patients were male. Prevalence of hypertension (57.2% vs. 30.6%, p=0.002) and smoking (70.6% vs. 52.8%, p=0.049) was significantly higher in ACS patients than in no-CAD patients. Prevalence of hypertension (72.1% vs. 38.6%, p<0.001) and body mass index (BMI) were significantly higher in MVD group than in SVD group. Multivariable analysis revealed that hypertension was an independent risk factor for MVD after adjustment for age, gender, BMI, smoking, family history of premature CAD, hyperlipidemia, left ventricular ejection fraction, and brain natriuretic peptide (odds ratio=3.71, 95% confidence interval=1.84-7.46, p<0.001). Rate of major adverse cardiovascular events (MACE) during follow-up (20.2% vs. 4.8%) was significantly higher in MVD group compared with SVD group.

**Conclusions:**

Hypertension is an independent predictor of MVD and MVD is associated with increased MACE rate compared to SVD in young ACS patients during the short-term follow-up.

## 1. Introduction

Risk factor profiles, clinical presentations, and prognosis might differ between young patients with acute coronary syndrome (ACS) and elderly ACS patients [1–4]. Previous studies showed that the prevalence of ACS among population less than 45 years of age (young ACS) ranged from 2% to 10% [4–6]. Young ACS cases were more prevalent among the Malays (49.8%), followed by Indians (24.4%), Chinese (21.8%), and other races (4.1%) [2]. Risk factors of ACS are age-dependent. Jamil et al. reported that prevalence of smoking (79.2% vs. 66.2%, p<0.001) was significantly higher, while prevalence of diabetes (12.1% vs. 25.6%, p<0.001), hypertension (34.4% vs. 57.4%, p<0.001), and hyperlipidemia (39.7% vs. 50.1%, p<0.001) was significantly lower in young ACS patients compared to elderly (>55 years old) ACS patients [7]. Smoking was identified as one of the major risk factors of ACS in young adults [2].

Several randomized controlled trials hinted that multivessel coronary artery disease (CAD) may occur in up to 50% of all CAD patients [8, 9]. Previous studies also demonstrated that patients with multiple-vessel disease (MVD) faced substantially increased risks of mortality and major adverse cardiac events, such as reinfarction or need for urgent revascularization after successful primary percutaneous coronary intervention (PCI) [10, 11]. It is known that incidence of diabetes, advanced age, impaired left ventricular function, and history of stroke are usually high in MVD patients [12, 13].

At present, there are only scanty reports on the prevalence and risk factors as well as outcome of MVD in young ACS patients. In the present study, we compared the risk factors and short-term outcome between young ACS patients with single-vessel disease (SVD) or MVD.

## 2. Methods

### 2.1. Study Population

In total, 187 consecutive young male adult (aged ≤45 years) ACS patients hospitalized in our department between January 2012 and December 2016 were enrolled in this study. Thirty-six young male patients with normal coronary angiography (CAG) findings (no-CAD), who underwent CAG due to suspected chest pain in this period, served as control group. The study was conducted in accordance with the Declaration of Helsinki and approved by the local Ethics Committee. Written informed consent was obtained from all patients.

ACS refers to any group of clinical symptoms compatible with acute myocardial ischemia and includes unstable angina (UA), non-ST-segment elevation myocardial infarction (NSTEMI), and ST-segment elevation myocardial infarction (STEMI). UA was defined as angina pectoris or equivalent ischemic discomfort with at least one of three features: (1) it occurs at rest (or with minimal exertion), usually lasting >10 minutes; (2) it is severe and of new onset (i.e., within the prior 4-6 weeks); (3) it occurs with a crescendo pattern (i.e., distinctly more severe, prolonged, or frequent than previously) [14]. STEMI was defined as the presence of typical chest pain accompanying symptoms for a duration of at least 30 minutes but <12 hours in the presence of ST-segment elevation ≥1 mm in at least 2 contiguous leads, or new or undetermined duration of left bundle branch block in association with elevated cardiac enzymes [creatine kinase myocardial band (CK-MB) and Troponin I] [15]. NSTEMI was defined as ECG ST-segment depression or prominent T-wave inversion and/or positive biomarkers of necrosis in the absence of ST-segment elevation and in an appropriate clinical setting (chest discomfort or angina equivalent) [15]. SVD referred single-vessel lumen stenosis ≥50%, luminal stenosis of left main coronary artery greater than 50%; MVD referred at least two main arteries with stenosis of vessel lumen ≥50%, luminal stenosis of left main coronary artery >50% by CAG [16, 17]. The degree of coronary artery stenosis was visually rated by 2 experienced interventional cardiologists. Hypertension was defined as a history of systolic blood pressure (SBP) ≥140mmHg or a diastolic blood pressure (DBP) ≥90mmHg or documented hypertension on at least two occasions in outpatient clinics or known hypertension under antihypertensive medication regardless of the current blood pressure [18]. Smoking was classified into three categories: never smokers, ex-smokers (those who had smoked regularly but had stopped smoking at least six months before the survey), and current smokers. We used the 2016 American Diabetes Association (ADA) guidelines for the diagnosis of diabetes [19], and the 2013 ACC/AHA guidelines management of dyslipidemias for the diagnosis of hyperlipidemia [20].

Procedural factors recorded included the infarct-related artery, number of diseased vessels, number of stents, and thrombus aspiration (TA) device use. Apart from the patient's baseline characteristics (ECG recordings, age, sex, hypertension, smoking status, hyperlipidemia, and family history of premature CAD and history of previous ACS), the following biochemical indices were analyzed: CK-MB, complete lipid profile, blood cell count, urea, creatinine, brain natriuretic peptide (BNP), and hepatic aminotransferases. Echocardiography was performed in all patients after CAG and/or PCI. All patients were followed up and treated according to the current guidelines of ACS [21].

### 2.2. Outcomes

The primary clinical outcome was major adverse cardiac events (MACE), defined as all-cause mortality, recurrent MI, stroke, coronary artery bypass graft (CABG), and repeat PCI during the follow-up period. Secondary clinical outcomes included in-hospital and 30 days all-cause mortality rate. The follow-up was made by clinical visit or telephone calls.

### 2.3. Statistical Analysis

Continuous variables are expressed as mean ± SD and categorical variables as number (percent). The data were analyzed by homogeneity of variances test. Continuous data with normal distribution were assessed by Student's* t*-test or one-way ANOVA with post hoc test (Bonferroni) as indicated. Nonnormal distribution data were tested by two-tailed Mann–Whitney U test or Kruskal-Wallis nonparametric test as indicated. Categorical data were compared across groups using Chi-square test or Fisher's exact test as appropriate. The associations of hypertension with MVD were evaluated using univariate and multivariate binary logistic regression models. Odds ratio (OR) and 95% confidence interval (CI) for MVD were calculated. In the multivariate models, age, gender, body mass index (BMI), hyperlipidemia, smoking, and family history of premature coronary artery disease, albumin, BNP, and left ventricular ejection fraction (LVEF) were included as covariates. P value <0.05 (two-tailed test) was considered statistically significant. The statistical analysis was performed using the SPSS statistical software, version 23.0 (IBM SPSS Statistics, Chicago, USA).

## 3. Results

### 3.1. Patient Characteristics

Table 1 shows the patient characteristics. All subjects are male. The median age of the MVD group was significantly older than that of control group (p=0.024). Hypertension was diagnosed in 118 out of 233 subjects (53%). Forty-four hypertensive patients received antihypertensive medication and the rest received no antihypertensive medication, and blood pressure was controlled in 19 out of 44 (43.2%) treated hypertensive patients. Hypertension and smoking were more frequent in the ACS group compared with no-CAD group (57.2% vs. 30.6%, p=0.002, and 70.6% vs. 52.8%, p=0.049, respectively). Regional wall motion abnormality was present in 50.8% of ACS patients. The prevalence of hypertension was significantly higher in MVD group than in SVD group (72.1% vs. 38.6%, p<0.001).

Table 2 presents the laboratory findings. WBC count, CK-MB, myoglobin, and high-sensitivity troponin I levels were significantly higher in ACS group than in no-CAD group and were similar between SVD and MVD groups. Prevalence of hyperlipidemia was low in this cohort (0% in no-CAD group and 3.2% in ACS group (p>0.05). Red blood cell count, platelet count, BNP, total cholesterol, triglyceride, low-density lipoprotein cholesterol, glucose, blood urea nitrogen, and uric acid level were similar among the three groups (Table 2).

### 3.2. Procedural and Coronary Artery Involvement Characteristics

**Figure 1:**
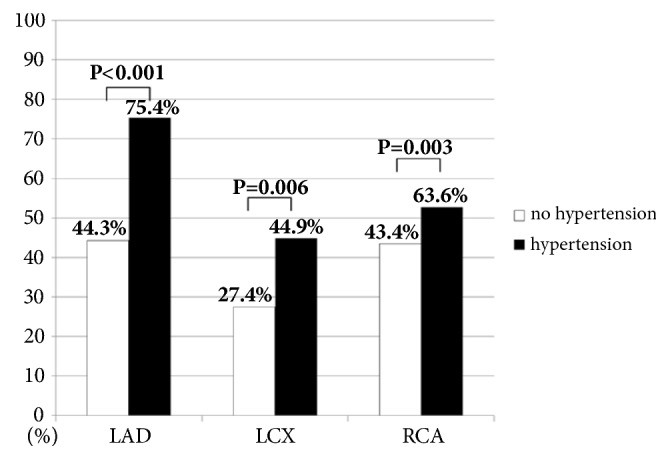
**The prevalence of involved vessels in the patients with or without hypertension**. Note that the prevalences of LAD-, RCA-, and LCX-related stenosis in patients with hypertension were higher than those in patients without hypertension. Additionally, in patients with hypertension, the LAD-related stenosis was more common compared with RCA- and LCX-related stenosis, p<0.001. LAD: left anterior descending artery; LCX: circumflex artery; RCA: right coronary artery.

Angiographic and procedural characteristics of the study population were li*s*ted in Table 3. The prevalences of left anterior descending artery (LAD), circumflex artery (LCX), and right coronary artery (RCA) lesion in the MVD group were significantly higher than in the SVD group. As shown in Figure 1, hypertension is related to higher prevalence of LAD, LCX, and RCA lesion. Additionally, prevalence of LAD-related stenosis (75.4%) was significantly more common than that of RCA (63.6%) and LCX-related stenosis (44.9%) in patients with hypertension (both p<0.001, Figure 1).

### 3.3. Hypertension and Smoking Are Independent Risk Factors for ACS in Young Adults

Table 4 shows the binary logistic regression results for ACS. Hypertension served as an independent risk factor for ACS (unadjusted OR 3.16, 95% CI 1.48-6.78, p=0.003), after adjustment for age, gender, and BMI (OR 2.91, 95% CI 1.30-6.52, p=0.009) and after adjustment for age, gender, BMI, smoking, family history of premature coronary artery disease, and hyperlipidemia (OR 3.42, 95% CI 1.48-7.88, p<0.001). Smoking is also an independent risk factor for ACS (unadjusted OR 2.04, 95% CI 0.99-4.19, p=0.052), after adjustment for age, gender, and BMI (OR 2.36, 95% CI 1.12-4.96, p=0.024) and after adjustment for age, gender, BMI, hypertension, family history of premature coronary artery disease, and hyperlipidemia (OR 2.49, 95% CI 1.16-5.34, p=0.019). BNP and LVEF were associated with the prognosis of ACS patients. After adding these two indexes as adjusted cofounders, the predicting efficacy of hypertension and smoking weakened to the borderline significant level: hypertension (p=0.077) and smoking (p=0.071).

### 3.4. Hypertension Is an Independent Risk Factor for MVD in Young ACS Patients

**Figure 2:**
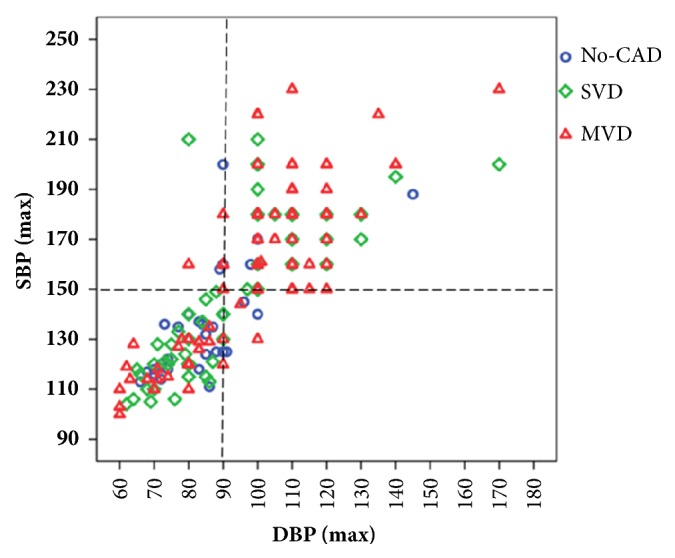
**Scatter plot of SBP and DBP among patients with no-CAD, SVD, and MVD**. Note that incidence of hypertension [SBP > 150mmHg (y-axis) and/or DBP > 90mmHg (x-axis)] was significantly higher in MVD patients (75 out of 104, 72.1%) than in patients with SVD (34 out of 83, 40.5%) and in no-CAD patients (16 out of 37, 44.4%). DBP: diastolic blood pressure; MVD: multivessel coronary artery disease, no-CAD: no coronary artery disease; SBP: systolic blood pressure; SVD: single-vessel disease.

Table 5 shows the binary logistic regression results for MVD. Hypertension remained as an independent risk factor for MVD (unadjusted OR 4.20, 95% CI 2.27-7.77, p<0.001) after adjustment for age, gender, and BMI (OR 3.59, 95% CI 1.89-6.83, p<0.001); after adjustment for age, gender, BMI, smoking, family history of premature coronary artery disease and hyperlipidemia (OR 3.63, 95% CI 1.88-7.01, p<0.001); after adjustment for age, gender, BMI, BNP, and albumin (OR 3.96, 95% CI 1.96-7.99, p<0.001); and after adjustment for age, gender, BMI, albumin, BNP, and LVEF (OR 3.71, 95% CI 1.84-7.46, p<0.001). As shown in Figure 2, incidence of hypertension [SBP >150mmHg and/or DBP >90mmHg] was 72.1% in MVD group, 40.5% in SVD group, and 44.4% in no-CAD group (p<0.001). Patients with SBP >150mmHg and/or DBP >90mmHg were significantly associated with MVD in this cohort (sensitivity 72% and specificity 58%).

### 3.5. In-Hospital and 30-Day Clinical Outcome

In-hospital MACE rates were 0.0% in the SVD and MVD groups; the 30-day MACE rate was 0.0% in SVD group and 1% (n=1, death) in MVD group (p=0.37).

### 3.6. Short-Term Clinical Outcome

The mean follow-up time was 267±124 days. MACE rate was significantly higher in MVD group (20.2%, 18 repeat PCI and 3 CABG) compared with SVD group (4.8%, 4 repeat PCI, p=0.002). There was no death during the follow-up period in this patient cohort. There was no significant difference between SVD and MVD groups in the rates of recurrent MI [1.2% (n=1) vs. 1.9% (n=2), p=0.698], stroke (0.0% vs. 0.0%), and CABG [0.0% (n=0) vs. 2.9% (n=3), p=0.119] during the follow-up period.

## 4. Discussion

To the best of our knowledge, this is the first study to evaluate the association between hypertension and MVD in young ACS patients. The major findings of the present study are as follows: Firstly, the presence of hypertension, but not smoking, is an independent predictor of MVD in young patients with ACS. Secondly, the rate of MACE was significantly higher in MVD group compared with SVD group during the 267±124 days of follow-up. Our results thus highlight the role of hypertension in the pathogenesis of MVD in young ACS patients, suggesting that hypertension control serves as an important strategy for the prevention and treatment of MVD in young ACS patients.

### 4.1. Risk Factors of ACS in Young Adults

Previous investigations have reported that young ACS patients have a different risk factor profile compared with elderly ACS patients [7, 22–24]. Hypertension is a known important risk factor for the development of coronary artery disease [25]. The impact of smoking on elderly patients with coronary artery disease is well established, while conflicting results existed on the impact of smoking in young adults with coronary artery disease [25, 26]. It was reported that the prevalence of hypertension was 25% in young coronary artery disease patients as compared to 13% in young non-coronary artery disease subjects and the prevalence of hypertension was much higher in elderly individuals with coronary artery disease than in young coronary artery disease patients [27]. In this study, we showed that prevalence of hypertension in young ACS patients was higher than previously reported and hypertension was more frequent in the ACS group compared with the no-CAD group (57.2% vs. 30.6%, p=0.002) and hypertension, together with smoking, served as independent risk factors for ACS. Conflicting results were reported on the impact of diabetes in young ACS patients [25, 26]. There is no diabetic patients in our real-world-derived patient cohort, there was also no young female ACS patients in our cohort, and the contribution of diabetes and gender effect could thus not be evaluated based on our data. Our study found that hypertension and smoking are the major risk factors of young male ACS patients, while hyperlipidemia and family history of coronary artery disease played only a negligible role in young male ACS patients based on data from this patient cohort.

### 4.2. Association between Hypertension and MVD in Young ACS Patients

The association between hypertension and MVD in young ACS patients remains controversial. Sukhija et al. observed higher prevalence of MVD in hypertensive patients compared to nonhypertensives [27]. However, Zand Parsa et al. did not find any relationship between hypertension and MVD [28]. Our results indicated a strong association between hypertension and MVD in young male ACS patients, in that the prevalence of hypertension is as high as 72.1% in MVD group compared to 38.6% in SVD group (p<0.001, Figure 2). Moreover, results of the ordinal logistic regression model for MVD revealed that hypertension was a significant independent risk factor for MVD after adjustment for smoking, BMI, family history of premature CAD, BNP, LVEF, and hyperlipidemia in young male ACS patients. In addition, our results suggested that SBP >150mmHg and/or DBP >90mmHg as the cut-off value could fairly predict the presence of MVD (sensitivity of 72% and specificity of 58%) in young male ACS patients.

### 4.3. Smoking and Prevalence of ACS and MVD in Young Adults

Previous studies have demonstrated that smoking is the most important risk factor associated with the severity of coronary artery disease and is significantly linked with increased risk of coronary plaque vulnerability, myocardial infarction, and cardiovascular death [29, 30]. Previous report showed that the prevalence of smoking in younger coronary artery disease individuals (<45 years of age) ranged from 60% to 90% as compared to 24% to 56% in subjects aged 45 years and over [31, 32]. In addition, smoking served as the most important modifiable risk factor for young adult patients with ACS [24]. Our data are in accordance with previous findings in that the prevalence of smoking was high (70.6%) in young ACS patients and smoking was an independent predictor of ACS in young adults [OR: 2.49 (95% CI 1.16-5.34)] after adjustment for age, gender, BMI, hyperlipidemia, hypertension, and family history of premature CAD (Table 4). However, smoking was not an independent risk factor after adding BNP and LVEF as adjusted cofounders for ACS, and smoking was not an independent risk factor for MVD.

### 4.4. Outcome of Young MVD Patients

Previous studies demonstrated that MVD was associated with worse prognosis compared to SVD patients [10, 33]. In this study, the in-hospital and 30-days MACE rates were similarly low in SVD group and MVD group (in-hospital MACE rate was both 0.0% in SVD and MVD groups and the 30-day MACE rate was 0.0% in SVD group and 1% in MVD group). During the short-term follow-up, there was no record on recurrent MI and stroke in young ACS patients. As expected, the rate of MACE rate was significantly higher in MVD group (20.2%, 18 repeat PCI and 3 CABG) than in SVD group (4.8%, 4 repeat PCI, p=0.002). It is to note that the relatively low in-hospital and 30-day MACE rate as well as the low MACE rate during the short-term follow-up period from patients in this cohort might be partly due to the use of new-generation drug-eluting stents. Recent studies suggested that stent thrombosis is less frequent with newer drug-eluting stents as compared to bare metal stents [34–36].

## 5. Limitations

The current study has several limitations. First, it was a retrospective and nonrandomized single-center study and caution is thus needed to extrapolate present study results to general young ACS and MVD population. Second, the relatively small patient cohort number serves as another study limitation. Third, there was no young female ACS patient in this cohort; this might relate to lower prevalence of ACS in young female population in our region; there is also no diabetic patient in our patient cohort. Therefore, our results could not be used to evaluate the contributing impact of diabetes and female gender on the pathogenesis of ACS and coronary vessel lesion, as well as outcome in young adults. Nevertheless, our patients are consecutive homogeneous unselected young patients with ACS; therefore, our data might exactly mirror the real-world scenario of young ACS as well as MVD patients in our region.

## 6. Conclusions

Hypertension serves as an independent risk factor of MVD and related to higher MACE rate during the short-term follow-up (death, repeat PCI, and CABG) in young male adults with ACS. Our results thus highlight the role of hypertension in the pathogenesis of MVD in young male ACS patients, indicating that rigorous hypertension control might be an important strategy for the prevention and treatment of MVD in young male ACS patients.

## Figures and Tables

**Table 1:** Clinical characteristics.

	**No-CAD **	**ACS**	
		SVD	MVD
	N=36	N=83	N=104
Age (years)	41 (37-43)	40 (38-44)	42 (40-45)*∗*
Gender (M/F)	36/0	83/0	104/0
BMI (kg/m^2^)	26.8±4.8	26.2±3.5	27.8±3.6^†^
Baseline SBP (mmHg)	131.5±14.2	127.0±20.1	128.6±19.4
Baseline DBP (mmHg)	81.6±9.0	78.9±13.5	80.3±15.1
HR (beats/min)	70.6±15.4	70.7±12.9	72.0±14.4
Hypertension [n (%)]	11 (30.6)	32 (38.6)	75 (72.1)*∗*^†^
Duration (years)	5.7±3.8	4.8±4.5	6.9±5.4
Family history [n (%)]	6 (16.9)	11 (13.3)	21 (20.2)
Smoking [n (%)]	19 (52.8)	64 (77.1)*∗*	68 (65.4)*∗*
Duration (year)	16.5±7.9	17.9±6.6	18.9±7.9
Consumption (cigarettes/day)	19.7±12.7	22.3±10.5	24.9±13.3
Alcohol use [n (%)]	6 (16.7)	14 (16.9)	13 (12.5)
Duration (year)	12.5 (10-20)	20 (17.5-20)	20 (10-20)
Consumption (g/day)	64 (20-103)	75 (27-150)	20 (20-75)
Family history of premature CAD [n (%)]	5 (13.9)	15 (18.1)	29 (27.9)
Diabetes mellitus [n (%)]	0	0	0
Hyperlipidemia [n (%)]	0	3 (3.6)	3 (2.9)
Echocardiography			
LVEF (%)	62.6±2.7	59.7±6.7	58.2±6.4*∗*
Regional wall motion abnormality [n (%)]	0	41 (49.4)*∗*	53 (52.0)*∗*

p<0.05 vs. no-CAD group;  ^†^p<0.05 vs. SVD group. ACS: acute coronary syndrome; BMI: body mass index; CAD: coronary artery disease; DBP: diastolic blood pressure; LVEF: left ventricular ejection fraction; MVD: multivessel coronary artery disease; SBP: systolic blood pressure; SVD: single-vessel disease.

**Table 2:** Laboratory findings.

	**No-CAD**	**ACS**	
		SVD	MVD
	N=36	N=83	N=104
WBC count (10^9^/L)	6.2 (5.3-7.5)	11.7 (6.8-14.6)*∗*	12.3 (6.6-14.6)*∗*
Hemoglobin (g/L)	151.5±11.4	149.4±13.9	153.2±16.5
Platelet count (10^9^/L)	209.7±56.7	217.9±45.6	221.5±50.7
CK-MB (ng/mL)	1.12 (0.9-2.7)	30 (2.3-52.0)*∗*	31 (2.4-56.0)*∗*
Myoglobin (ng/mL)	34.0 (23.5-53.0)	439.7 (35.0-500)*∗*	426.5 (35.3-558.2)*∗*
High-sensitivity troponin I (ng/mL)	0.08 (0.05-0.29)	5.2 (0.1-6.8)*∗*	5.2 (0.3-8.6)*∗*
BNP (pg/ml)	17.3 (8.7-40.6)	40 (17.3-82.3)*∗*	60 (26.2-127.5)*∗*
Total protein (g/L)	68.2±6.4	63.0±7.8*∗*	62.9±6.6*∗*
Albumin (g/L)	42.4±4.1	39.2±5.1*∗*	39.2±4.5*∗*
Globulin (g/L)	24.7±4.5	25.6±3.8	25.6±3.7
A/G	1.7±0.3	1.8±2.7	1.6±0.4
TG (mmol/L)	1.3 (0.9-1.8)	1.5 (1.2-2.0)	2.5 (1.4-2.5)*∗*
TC (mmol/L)	4.4±1.2	4.5±1.3	4.7±1.3
HDL-C (mmol/L)	1.2±0.3	1.1±0.3	1.1±0.2
LDL-C (mmol/L)	2.4±1.0	2.8±1.0	2.8±1.0
GLU (mmol/L)	5.1±0.8	5.4±1.4	5.5±1.2
BUN (mmol/L)	5.3±1.0	5.1±1.3	5.1±1.4
SCr (*µ*mol/L)	89.2±14.1	87.5±17.2	90.7±15.5
Uric Acid (*µ*mol/L)	331.8±127.5	349.6±115.9	370.9±93.6

*∗*p<0.05 vs. no-CAD group,  _ _^†^p<0.05 vs. SVD group. ACS: acute coronary syndrome; A/G: albumin to globulin ratio; BNP: brain natriuretic peptide; BUN: blood urea nitrogen; CAD: coronary artery disease; CK-MB: creatine kinase myocardial band; GLU: glucose; HDL-C: high-density lipoprotein cholesterol; LDL-C: low-density lipoprotein cholesterol; MVD: multivessel coronary artery disease; RBC: read blood cell; SCr: serum creatinine; SVD: single vessel disease; TC: total cholesterol; TG: triglyceride; WBC: white blood cell.

**Table 3:** Angiographic and procedural characteristics.

	**No-CAD **	**ACS**	
		SVD	MVD
	N=36	N=83	N=104
Stenosis-related artery LM [n (%)]	0	1 (1.2)	3 (2.9)
Stenosis-related artery LAD [n (%)]	0	40 (48.2)*∗*	96 (92.3)*∗*^†^
Stenosis-related artery LCX [n (%)]	0	11 (13.3)*∗*	71 (68.9)*∗*^†^
Stenosis-related artery RCA [n (%)]	0	31 (37.3)*∗*	90 (86.5)*∗*^†^
TA device used [n (%)]	0	6 (7.2)	7 (6.7)
Number of stents	0	0.8±0.7*∗*	1.1±0.9*∗*^†^
Prior MI [n (%)]	0	0	0
Prior PCI [n (%)]	0	0	0
Prior CABG [n (%)]	0	0	0

*∗*p<0.05 vs. no-CAD group,  ^†^p<0.05 vs. SVD group. ACS: acute coronary syndrome; CABG: coronary artery bypass graft; CAD: coronary artery disease; LAD: left anterior descending artery; LCX: circumflex artery; LML left main; MIL myocardial infarction; MVD: multivessel coronary artery disease; PCI: percutaneous coronary intervention; RCA: right coronary artery; SVD: single-vessel disease; TA: thrombus aspiration.

**Table 4:** Hypertension and smoking for prediction of acute coronary syndrome based on multivariable logistic regression models (n=223).

	Unadjusted OR	95% CI	p value

Hypertension	3.16	1.48-6.78	0.003
Smoking	2.04	0.99-4.19	0.052

	Adjusted OR	95% CI	p value

Hypertension	2.91	1.30-6.52	0.009
(Adjusted for age, gender, and BMI)			

Hypertension	3.42	1.48-7.88	<0.001
(Adjusted for age, gender, BMI, hyperlipidemia, smoking, and family history of premature CAD)			

Hypertension	2.94	0.89-9.73	0.077
(Adjusted for age, gender, BMI, hyperlipidemia, smoking, family history of premature CAD, BNP, and LVEF)			

Smoking	2.36	1.12-4.96	0.024
(Adjusted for age, gender, and BMI)			

Smoking	2.49	1.16-5.34	0.019
(Adjusted for age, gender, BMI, hyperlipidemia, hypertension, and family history of premature CAD)			

Smoking	2.72	0.92-8.07	0.071
(Adjusted for age, gender, BMI, hyperlipidemia, hypertension, family history of premature CAD, BNP, and LVEF)			

BMI: body mass index; BNP: brain natriuretic peptide; CAD: coronary artery disease; CI: confidence interval; LVEF: left ventricular ejection fraction; OR: odds ratio.

**Table 5:** Hypertension for prediction of multivessel coronary artery disease based on multivariable logistic regression models (n=187).

	Unadjusted OR	95% CI	p value

Hypertension	4.20	2.27-7.77	<0.001

	Adjusted OR	95% CI	p value

Hypertension	3.59	1.89-6.83	<0.001
(Adjusted for age, gender, and BMI)			

Hypertension	3.63	1.88-7.01	<0.001
(Adjusted for age, gender, BMI, hyperlipidemia, smoking, and family history of premature CAD)			

Hypertension	3.71	1.84-7.46	<0.001
(Adjusted for age, gender, BMI, albumin, BNP, and LVEF)			

BMI: body mass index; BNP: brain natriuretic peptide; CAD: coronary artery disease; CI: confidence interval; LVEF: left ventricular ejection fraction; OR: odds ratio.

## Data Availability

The data used to support the findings of this study are included within the article. The other data used to support the findings of this study are available from the corresponding author upon request.
